# Establishing thresholds of handgrip strength based on mortality using machine learning in a prospective cohort of Chinese population

**DOI:** 10.3389/fmed.2023.1304181

**Published:** 2023-12-01

**Authors:** Haofeng Zhou, Zepeng Chen, Yuting Liu, Yingxue Liao, Lan Guo, Mingyu Xu, Bingqing Bai, Fengyao Liu, Huan Ma, Xiaoxuan Yao, Qingshan Geng

**Affiliations:** ^1^Guangdong Cardiovascular Institute, Guangdong Provincial People’s Hospital, Guangdong Academy of Medical Sciences, Guangzhou, China; ^2^Shantou University Medical College, Shantou, China; ^3^Department of Cardiology, Shenzhen People's Hospital and The First Affiliated Hospital, Southern University of Science and Technology, Shenzhen, China; ^4^School of Medicine, South China University of Technology, Guangzhou, China

**Keywords:** handgrip strength, threshold, health screening, mortality, machine learning

## Abstract

**Background:**

The relative prognostic importance of handgrip strength (HGS) in comparison with other risk factors for mortality remains to be further clarified, and thresholds used for best identify high-risk individuals in health screening are not yet established. Using machine learning and nationally representative data from the China Health and Retirement Longitudinal Study (CHARLS), the study aimed to investigate the prognostic importance of HGS and establish sex-specific thresholds for health screening.

**Methods:**

A total of 6,762 participants from CHARLS were enrolled. A random forest model was built using 30 variables with all-cause mortality as outcome. SHapley Additive exPlanation values were applied to explain the model. Cox proportional hazard models and Harrell’s C index change were used to validate the clinical importance of the thresholds.

**Results:**

Among the participants, 3,102 (45.9%) were men, and 622 (9.1%) case of death were documented follow-up period of 6.78 years. The random forest model identified HGS as the fifth important prognostic variable, with thresholds for identifying high-risk individuals were < 32 kg in men and < 19 kg in women. Low HGS were associated with all-cause mortality [HR (95% CI): 1.77 (1.49–2.11), *p* < 0.001]. The addition of HGS thresholds improved the predictive ability of an established office-based risk score (C-index change: 0.022, *p* < 0.001).

**Conclusion:**

On the basis of our thresholds, low HGS predicted all-cause mortality better than other risk factors and improved prediction of a traditional office-based risk score. These results reinforced the clinical utility of measurement of HGS in health screening.

## 1 Introduction

Despite remarkable progress achieved in recent decades, low- and middle-income countries still faces numerous health challenges, particularly in the context of population aging. The number of older adults individuals is expected to rise from 524 million in 2010 to nearly 1.5 billion in 2050, with most of the increase in low- and middle-income countries (1). Due to the rising prevalence of non-communicable diseases, environmental pollution and unhealthy lifestyles, many vulnerable adults are at high risk of death (2). However, owing to shortage of medical resources, implementing comprehensive health screening programs to identify high-risk individuals is of great difficulty in low- and middle-income countries (3). As a result, it is necessary to develop both affordable and easy methods to improve the effectiveness and coverage of screening.

Handgrip strength (HGS) is an important indicator of physical function, and a biological marker of aging throughout the lifespan (4). Extensive studies have consistently demonstrated a robust correlation between low HGS and a range of adverse health outcomes, including hip fracture, nutrition deficiencies, new-onset chronic diseases and mortality risk (5–9). The measurement of HGS involves a simple and highly reproducible procedure utilizing a portable and relatively low-cost dynamometer, so it can be easily implemented in regions with limited advanced medical equipment (10). Therefore, the measurement of HGS may be particularly relevant in low-resource setting to identify high-risk individuals.

However, the clinical utility of HGS as a screening tool is limited due to the lack of clarity regarding its predictive importance in comparison with other risk factors and the absence of data-driven thresholds for risk discrimination. Traditional regression analysis faces challenges in addressing this issue, mainly attributed to multicollinearity, which hampers the interpretability of results (11). In contrast, machine learning can handle multicollinearity, capture intricate relationships among variables, and pinpoint crucial variables contributing to the prediction models, with random forest is particularly useful in this context (12). Random forest allows for insights into the relative contribution of different variables towards the outcome by constructing an ensemble of decision trees and combines their predictions (13).

Utilizing the nationally representative data from the China Health and Retirement Longitudinal Study (CHARLS), The purpose of this study was to investigate the clinical utility of HGS in screening setting. We hypothesized that machine learning would provide novel insights into the relative importance of this data-driven approach HGS and establish clinically relevant thresholds for health screening.

## 2 Method

### 2.1 Study population

The CHARLS is a nationwide study aimed at analyzing the aging process and promoting interdisciplinary research in China. Representing samples aged 45 or older were selected from 450 villages and 150 districts in 28 provinces using multistage stratified Probability Proportionate to Size Sampling (14). Participants in the CHARLS completed a structured questionnaire about sociodemographic status, health status, physical function, and retirement information. 13 physical measurements and blood sample collection were also conducted. The baseline survey, referred to as wave 1, was conducted in 2011, with a response rate of 73.1%, and follow-ups were conducted every 2 years (wave 2 in 2013, wave 3 in 2015 and wave 4 in 2018). Ethical approval was obtained from the ethical review committee at Peking University (No. IRB 00001052-11,014), and all participants provided written informed consent before being surveyed. Detailed information on the CHARLS is available on the website: http://charls.Pku.edu.cn/.

**Figure 1:**
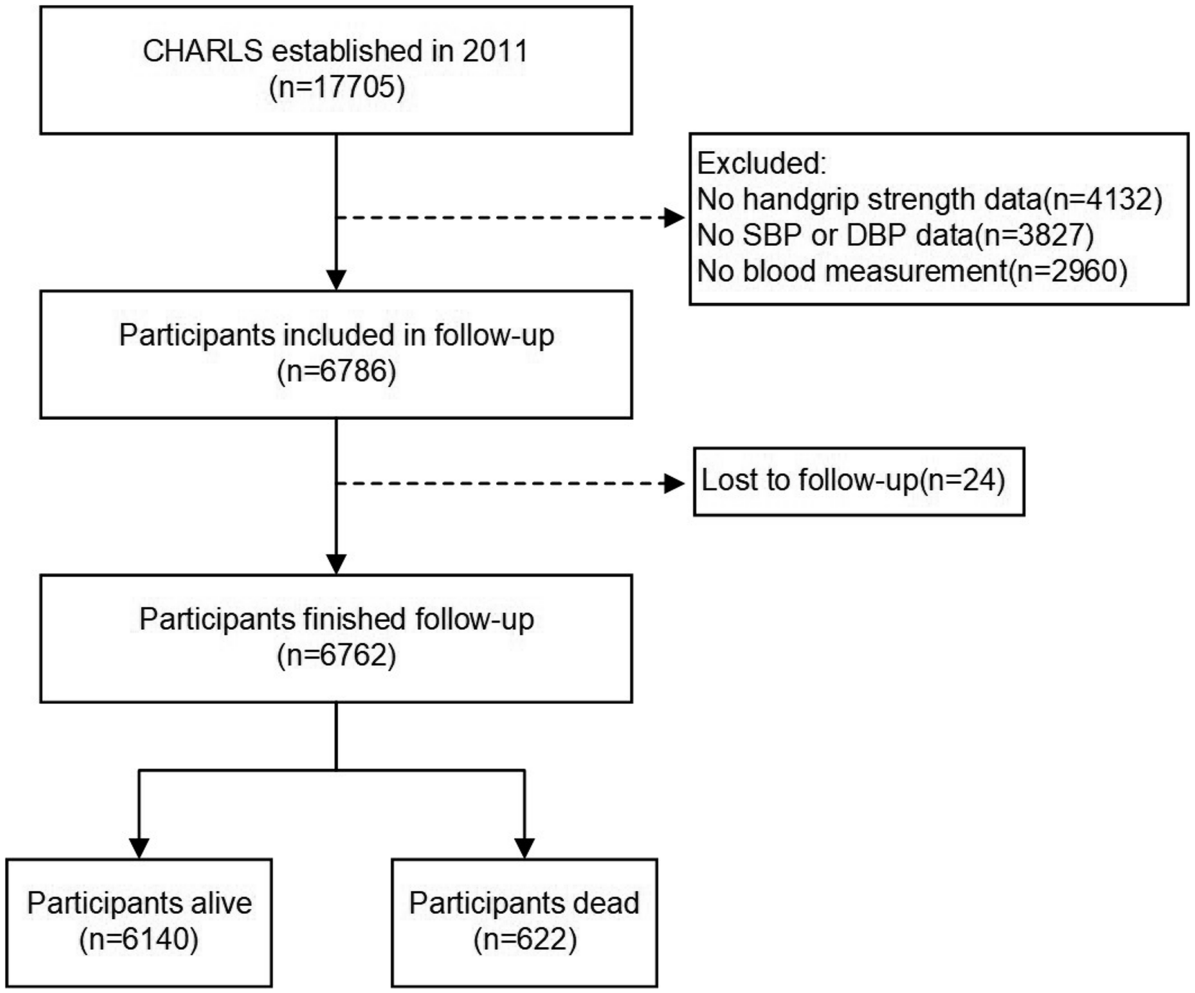
Flow diagram for participants included in the study.

In this study, we used baseline data from wave 1 and follow-up information from wave 2, 3 and 4. The exclusion criteria for the study were: missing baseline data of HGS, blood pressure (systolic or diastolic), or blood measurements in wave 1; and non-available follow-up information in wave 2,3 and 4. Of the total 17,705 participants in wave 1, 5,958 individuals were excluded because of missing data on HGS (*n* = 4,132), blood pressure (*n* = 3,827), or blood measurement (*n* = 2,960); 24 participants lost to follow-up were also excluded. As a result, a total of 6,762 participants were included in this study, with 622 experiencing death events during the follow-up period. The flow chart of the participant selection process is presented in Figure 1.

### 2.2 Measurement of HGS

HGS was measured by trained interviewers using a YuejianTM WL-1000 dynamometer with an accuracy of 0.1 kg. Before the measurement, interviewers would make sure that participants did not report any surgery, pain, inflammation, or severe injury to either of their hands. After interviewer providing a demonstration of how to use the dynamometer, participants were than instructed to squeeze the dynamometer as hard as possible for several seconds, with participants seated upright with their elbow by their side and flexed at a 90-degree angle. The measurement was repeated for participants’ right and left hands separately in two alternative turns, and the maximum value achieved during the process was recorded. For this study, the HGS value of dominant hand was used for analysis.

### 2.3 Assessment of outcome

The primary outcome of the study was all-cause mortality. The determination of death was based on the interview status (alive or dead) of participants during waves 2, 3, and 4 of the follow-up surveys. For participants who survived throughout the observation period, the survival time was determined as the interval between wave 1 and wave 4. In cases where death occurred, the survival time was calculated as the interval from the date of the baseline survey to the date of participants’ death. Because the exact death time was only available in wave 2, the death date of wave 3 and 4 was estimated based on the median time between the two waves when death recorded.

### 2.4 Variables used for the analysis

A total of 30 variables were included in the analysis (HGS, 6 demographic, 13 health-related, and 10 from blood measurements). Demographic characteristics included age, gender, residential area (rural or urban), education (no formal education, primary school, middle or high school, college or above), marital status (married or others) and medical insurance (yes or no). Health-related factors included body mass index (BMI), smoking and drinking (yes or no), systolic blood pressure (SBP), diastolic blood pressure (DBP), and self-reported physician-diagnosed chronic diseases (hypertension, diabetes, cardiovascular diseases, stroke, chronic lung diseases, chronic kidney diseases, liver disease, and cancers). Blood measurements included hemoglobin (Hb), hematocrit, mean corpuscular volume (MCV), total cholesterol, triglycerides, low-density lipoprotein cholesterol (LDL-C), high-density lipoprotein cholesterol (HDL-C), estimated glomerular filtration rate (eGFR), HbA1c, C-reactive protein (CRP). eGFR was calculated according to the Chronic Kidney Disease Epidemiology Collaboration’s 2009 creatinine equation (15).

### 2.5 Machine learning

Participants were randomly divided into a training cohort (70%; *n* = 4,733) and a validation cohort (30%; *n* = 2029), with a fixed random seed to ensure the repeatability of the experiment. The random forest model for predicting mortality was built with 30 variables using the training cohort. The model was evaluated and optimized using a 5-fold cross-validation method. The analysis involved using grid search to optimize the model and evaluating all available predictor variables for each split. In the optimal model of female population, a total of 10 independent trees were employed, with a maximum depth of 9, a minimum number of samples required for an internal node split equal to 2, and a minimum leaf size of 1. In the optimal model of male population, a total of 200 independent trees were used, with a maximum depth of 9, a minimum number of samples required for an internal node split equal to 2, and a minimum leaf size of 2. Models derived from the training cohort were then applied to the validation cohort to assess the predictive performance. The feature importance score from the random forest models were obtained to identify the predictive value of HGS on mortality. Accuracy and precision of each HGS value were calculated to determine optimal sex-specific thresholds with models. In addition, SHapley Additive exPlanation (SHAP) were used to provide attribution values of HGS within the random forest model (16). The SHAP summary plot illustrated the effect of different variables on the prediction, which displays the positive (red) and negative (blue) influences; the SHapley Additive exPlanation (SHAP) dependence plots, depicting HGS values on the horizontal axis and SHA*p* values (indicating influence) on the vertical axis, could reveal the impact of different HGS values on the mortality and discover linear or non-linear relationships.

### 2.6 Validation of the thresholds from the random forest model

We conducted the following analyzes to validate the clinical utility of HGS thresholds obtained from the random forest model: 1) conducting Kaplan–Meier survival analysis using the HGS thresholds and 2) verifying the incremental predictive information provided by the HGS thresholds when added to an established office-based risk score for predicting all-cause mortality (17).

### 2.7 Statistical analysis

Kolmogorov–Smirnov tested continuous variables for normality. Continuous variables with normal distribution were presented as mean and standard deviation (SD) and analyzed by the Student’s *t*-test and, while continuous variables with non-normal distribution were presented as median and interquartile range (IQR) and analyzed by the Mann–Whitney U test. Categorical data were presented as frequency and percentage and analyzed by the Chi-square test or Fisher exact test. Univariate linear regression analysis was conducted for evaluating the relationship between variables. Kaplan–Meier survival analysis was conducted to determine the difference of the survival rate between individuals above or below the HGS thresholds, compared using the log-rank test. The predictability of Cox models was calculated as Harrell’s C-index. To validate the predictive ability of HGS in different screening setting, we calculated the Harrell’s C-index in rural population and urban population separately. All statistical analyzes were performed using Python 3.9.12. A 2-sided *p* value <0.05 was considered statistically significant.

## 3 Result

### 3.1 Baseline characteristics of the study participants

A total of 6,762 participants were included in this study, and baseline characteristics are presented in the Table 1. The mean (SD) age of the study population was 58.8 (9.7) years, and 3,102 (45.9%) were male. The average HGS value was 31.8 (10.9) kg, and HGS exhibited a linear decline with increasing age (male: *r* = −0.49, female: *r* = −0.32), as presented in Supplementary Figure S1. Over a median follow-up period of 6.78 (range 3.49 to 7.25) years, a total of 622 (9.1%) cases of death were documented. Participants who died were older, predominantly male and not be married, living in an urban area and had a smoking habit. The dead participants had a higher prevalence of comorbidities, including hypertension, diabetes, cardiovascular disease, stroke, chronic lung disease, and cancer. Regarding blood measurement, participants who died had worse results. Importantly, those who died had lower HGS value than alive participants. Participants were randomly divided by a 7:3 ratio into a training cohort (*n* = 4,733) and a validation cohort (*n* = 2029). And clinical characteristics were comparable between groups (*p* > 0.05), as presented in (Supplementary Table S1).

**Table 1:** Baseline characteristics of the alive and dead participants.

Characteristics	Total (*n* = 6,762)	Alive (*n* = 6,140)	Dead (*n* = 622)	*p*-value
Age, y	58.8 (9.7)	57.9 (9.2)	67.4 (10.4)	<0.001
Female, *n* (%)	3,660 (54.1)	3,415 (55.6)	245 (39.4)	<0.001
Married (vs others)	5,683 (84.0)	5,245 (85.4)	438 (70.4)	<0.001
Rural (vs urban)	2,482 (36.7)	2,299 (37.4)	183 (29.4)	<0.001
Medical insurance, *n* (%)	6,327 (93.6)	5,750 (93.6)	577 (92.8)	0.442
Education				<0.001
No formal education	3,071 (45.4)	2,696 (43.9)	375 (60.3)	
Primary school	1,508 (22.3)	1,368 (22.3)	140 (22.5)	
Middle or high school	2098 (31.0)	1995 (32.5)	103 (16.6)	
College or above	85 (1.3)	81 (1.3)	4 (0.6)	
Smoking	2,660 (39.3)	2,316 (37.7)	344 (55.3)	<0.001
Drinking	1812 (26.8)	1,620 (26.9)	162 (26.0)	0.010
BMI	24.2 (31.3)	24.4 (32.2)	23.2 (21.2)	0.204
Blood pressure, mmHg				
Systolic	132.1 (31.8)	131.2 (30.4)	141.0 (42.7)	<0.001
Diastolic	75.8 (12.0)	75.8 (11.9)	75.9 (13.0)	0.840
Comorbidities, *n* (%)				
Hypertension	1821 (26.9)	1,595 (26.0)	226 (36.3)	<0.001
Diabetes	451 (6.7)	391 (6.4)	60 (9.6)	0.002
Cardiovascular disease	885 (13.1)	780 (12.7)	105 (16.9)	0.004
Stroke	173 (2.6)	136 (2.2)	37 (5.9)	<0.001
Chronic lung disease	645 (9.5)	532 (8.7)	113 (18.2)	<0.001
Chronic kidney disease	382 (5.6)	338 (5.5)	44 (7.1)	0.127
Liver disease	220 (3.3)	193 (3.1)	27 (4.3)	0.137
Cancers	58 (0.9)	43 (0.7)	15 (2.4)	<0.001
Blood measurements				
Hb, g/L	14.4 (2.1)	14.5 (2.1)	14.3 (2.3)	0.072
Hematocrit, %	41.8 (6.0)	41.9 (5.9)	41.1 (6.7)	0.004
MCV, fl	90.8 (8.3)	90.7 (8.2)	92.5 (9.0)	<0.001
Total cholesterol, mg/dL	193.7 (38.8)	194.0 (38.6)	190.2 (40.6)	0.026
Triglycerides, mg/dL	135.1 (106.1)	136.1 (107.5)	125.6 (89.9)	0.007
HDL-C, mg/Dl	50.7 (15.3)	50.7 (15.3)	51.6 (15.5)	0.145
LDL-C, mg/dL	117.2 (35.3)	117.6 (35.1)	114.0 (37.6)	0.023
eGFR, mL/min/1.73 m^2^	98.6 (25.1)	99.1 (24.9)	92.7 (26.2)	<0.001
HbA1c, mmol/mol	5.3 (0.8)	5.3 (0.8)	5.4 (1.2)	0.001
CRP, mg/L	2.7 (7.2)	2.4 (5.9)	6.1 (14.8)	<0.001
Handgrip strength, kg	31.8 (10.9)	32.2 (10.8)	28.1 (11.1)	<0.001

BMI, body mass index; Hb, hemoglobin; MCV, mean corpuscular volume; HDL, high density cholesterol; LDL, low density cholesterol; eGFR, estimated glomerular filtration rate; HbA1c, glycosylated hemoglobin; CRP=C-reactive protein.

### 3.2 Feature importance and thresholds of HGS

**Figure 2:**
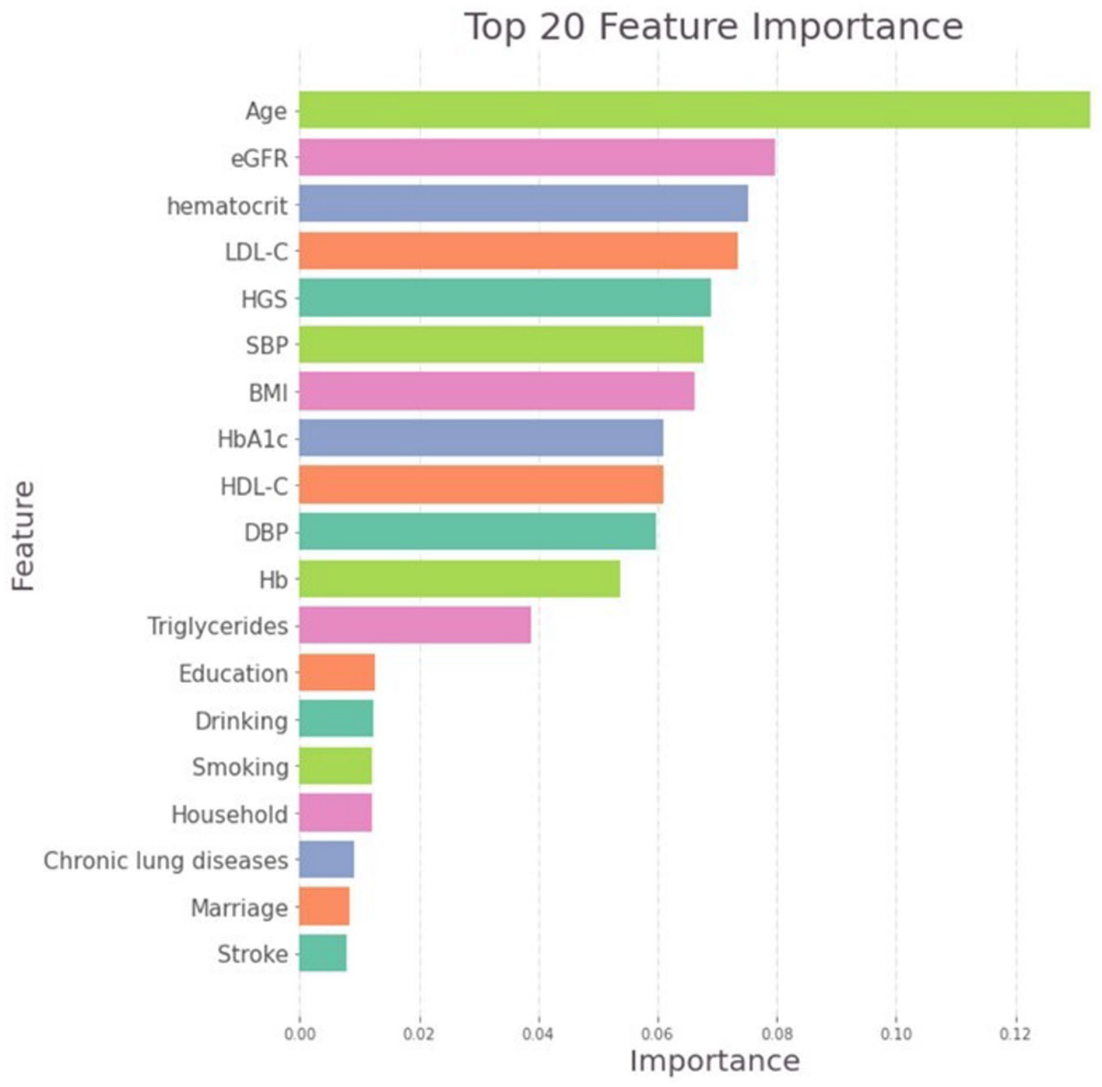
Importance matric plot of the random forest model.

In the random forest model, the most important variable contributing to mortality was age, followed by eGFR, hematocrit and LDL-C. Notably, HGS ranked as the fifth important variable and most predictive non-invasive variable (Figure 2), outperforming many traditional risk factors, including BMI, SBP and most chronic diseases. Based on the model performance, the sex-specific HGS thresholds for identifying high-risk individuals were < 19 kg in women (95% CI 19–20, accuracy = 0.94, precision = 0.94) and < 32 kg in men (95% CI 28–32, accuracy = 0.88, precision = 0.89). Given that age emerged as the most important factor in the random forest model, we also conducted a stratified analysis for different age groups using a moving window approach. The findings suggested that HGS thresholds manifested a decreasing pattern as the age range increased (Supplementary Figure S2).

### 3.3 Explanation of the random forest model

**Figure 3:**
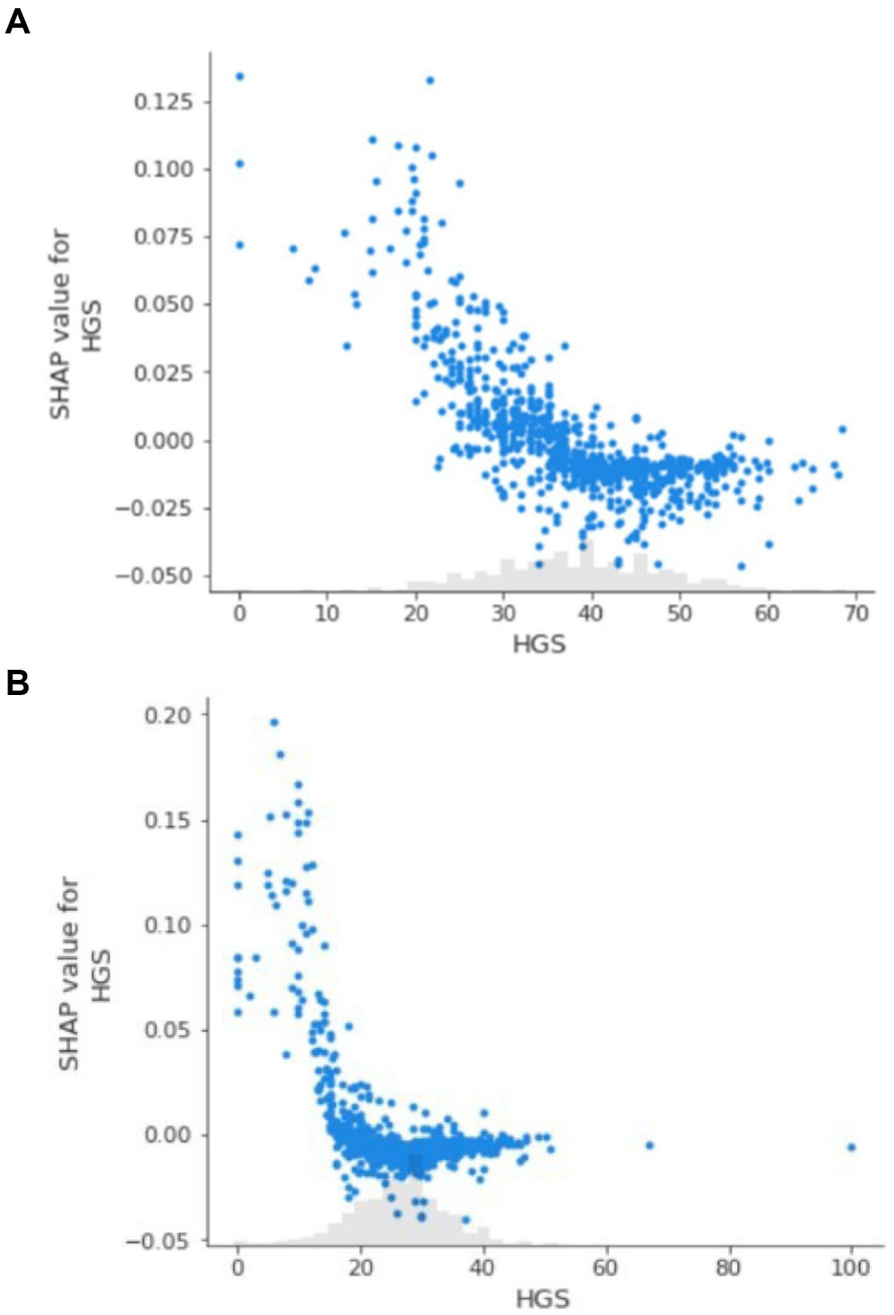
**(A)** SHAP dependence plot for handgrip strength in men. **(B)** SHAP dependence plot for handgrip strength in women.

The SHAP summary plot provided insights into the relative importance of variables included in the random forest model, and HGS exhibited the highest negative impact on the mortality (Supplementary Figure S3). SHAP dependence plots of HGS were constructed for men and women, respectively (Figure 3). On these plots, HGS demonstrated distinct nonlinear behaviors not previously apparent with the conventional regression analysis. Specifically, SHAP values were higher than zero (indicating higher mortality risk) when HGS were < 32 kg in men and < 19 in women, and exhibited decline as HGS increased; SHAP values reached a plateau once HGS exceeded the thresholds.

### 3.4 Baseline characteristics stratified by sex-specific HGS thresholds

Based on the determined thresholds, 1,281 patients were categorized as having low HGS. The comparison of baseline characteristics between low HGS and normal HGS groups is presented in Table 2. Low HGS was associated with old age, male, married status, living in the urban area, lower educational level, few medical insurances rate and previous smoking history. Participants with low HGS had lower BMI, higher SBP but lower SBP, and a high prevalence of most comorbidity. Furthermore, HGS showed significant relationships with various indices of blood measurement including MCV, serum lipid level, eGFR, HbA1c and CRP.

**Table 2:** Baseline characteristics of participants stratified by sex-specific HGS thresholds.

Characteristics	Low HGS (*n* = 1,281)	Normal HGS (*n* = 5,481)	*p* value
Age, y	65.7 (9.8)	57.2 (8.9)	<0.001
Female, *n* (%)	548 (42.8)	3,112 (56.8)	<0.001
Married (vs others)	285 (22.2)	794 (14.5)	<0.001
Rural (vs others)	422 (32.9)	2060 (37.6)	0.002
Medical insurance, *n* (%)	1,176 (91.8)	5,151 (94.0)	0.005
Education			<0.001
No formal education	747 (58.3)	2,324 (42.4)	
Primary school	291 (22.7)	1,217 (22.2)	
Middle or high school	234 (18.3)	1864 (34.0)	
College or above	9 (0.7)	76 (1.4)	
Smoking	622 (48.6)	2038 (37.2)	<0.001
Drinking	317 (24.7)	1,495 (27.3)	0.082
BMI	22.4 (3.9)	24.7 (34.7)	<0.001
Blood pressure, mmHg			
Systolic	133.7 (27.2)	131.8 (32.8)	0.031
Diastolic	74.4 (12.2)	76.2 (12.0)	<0.001
Comorbidities, *n* (%)			
Hypertension	400 (31.2)	1,421 (25.9)	<0.001
Diabetes	98 (7.7)	353 (6.4)	0.134
Cardiovascular disease	206 (16.1)	679 (12.4)	<0.001
Stroke	60 (4.7)	113 (2.1)	<0.001
Chronic lung disease	174 (13.6)	471 (8.6)	<0.001
Chronic kidney disease	94 (7.3)	288 (5.3)	0.005
Liver disease	53 (4.1)	167 (3.0)	0.058
Cancers	9 (0.7)	49 (0.9)	0.617
Blood measurements			
Hb, g/L	14.4 (2.2)	14.5 (2.1)	0.095
Hematocrit, %	41.9 (6.1)	41.8 (6.0)	0.353
MCV, fl	92.3 (8.4)	90.5 (8.3)	<0.001
Total cholesterol, mg/dL	188.4 (38.0)	194.9 (38.8)	<0.001
Triglycerides, mg/dL	123.6 (81.9)	137.8 (110.8)	<0.001
HDL-C, mg/dL	51.3 (15.5)	50.6 (15.2)	0.147
LDL-C, mg/dL	113.9 (34.2)	118.0 (35.6)	<0.001
eGFR, mL/min/1.73 m^2^	96.5 (24.7)	99.0 (25.1)	0.001
HbA1c, mmol/mol	5.3 (1.0)	5.3 (0.8)	0.004
CRP, mg/L	3.7 (9.2)	2.5 (6.7)	<0.001

HGS, handgrip strength; BMI, body mass index; Hb, hemoglobin; MCV, mean corpuscular volume; HDL, high density lipoprotein; LDL, low density lipoprotein; eGFR, estimated glomerular filtration rate; HbA1c, glycosylated hemoglobin; CRP, C-reactive protein.

### 3.5 Verification of the thresholds

**Figure 4:**
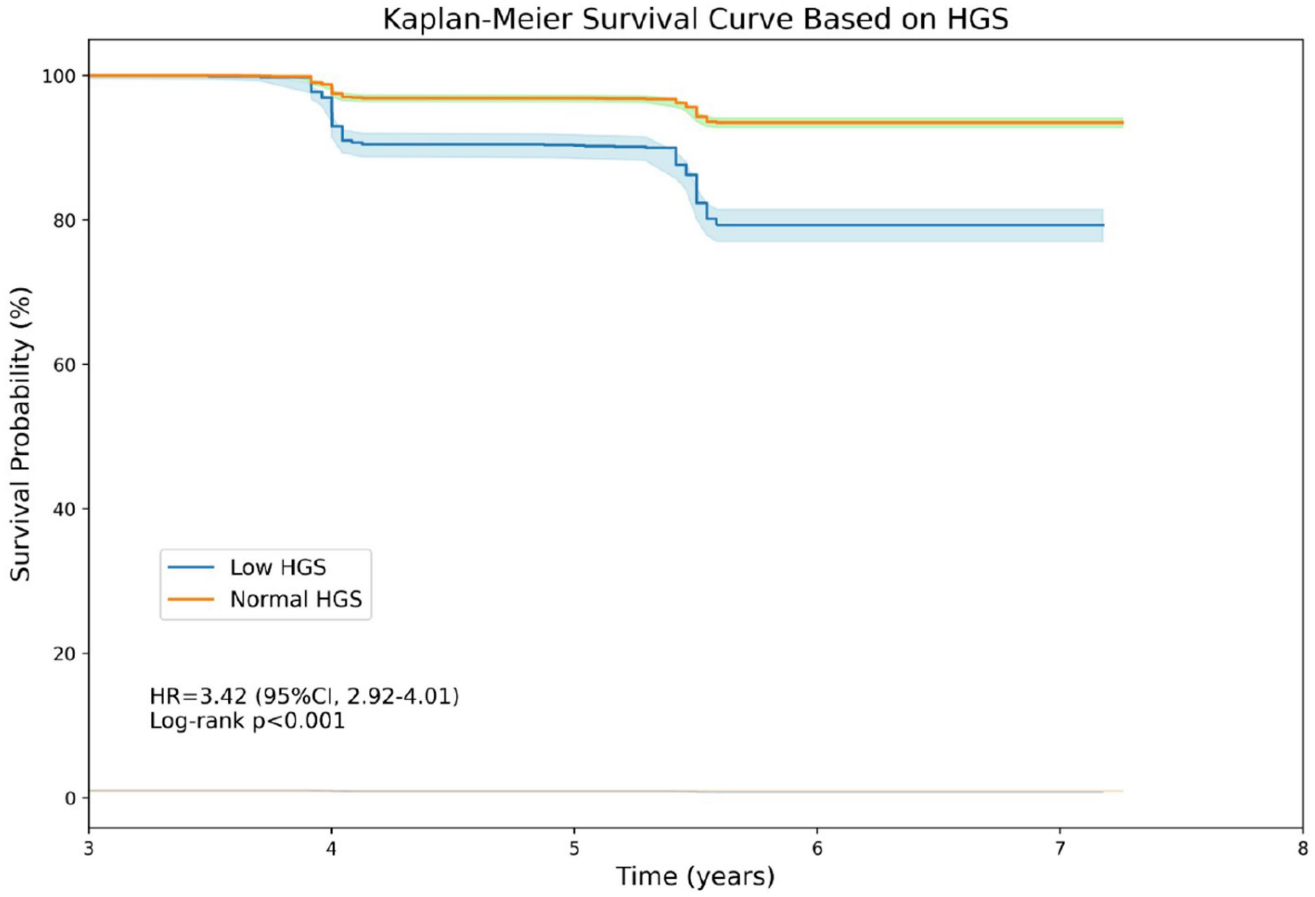
Kaplan-Meier survival curve based on handgrip strength.

Kaplan–Meier survival analysis was performed to investigate the relationship between HGS thresholds and all-cause mortality (Figure 4). Individuals with low HGS had a worse survival rate than those with normal HGS (HR:3.42, 95% CI: 2.92–4.01, *p* < 0.001). Results of additive predictive value provided by HGS thresholds is presented in Table 3. It is indicated that the office-based risk score (including age, sex, smoking, blood pressure, diabetes and BMI) had yielded a C-index of 0.720 (95% CI: 0.701–0.738) for all-cause mortality in all participants, and the C-index was 0.742 (95% CI: 0.725–0.760) after adding HGS thresholds, representing a marginal significant increase of 0.022 (95% CI: 0.020–0.024, *p* < 0.001). Similar improvement was observed in urban population and rural population, separately.

**Table 3:** Improvement in prediction when adding HGS thresholds to the office-based risk score.

	C index (95% CI)	C index change (95% CI)	*p* value
*All participants*			
Office-based risk score	0.720 (0.701–0.738)	0.022 (0.020–0.024)	<0.001
Plus HGS	0.742 (0.725–0.760)		
*Urban population*			
Office-based risk score	0.716 (0.609–0.738)	0.025 (0.021–0.029)	<0.001
Plus HGS	0.741 (0.723–0.759)		
*Rural population*			
Office-based risk score	0.724 (0.689–0.766)	0.019 (0.018–0.021)	<0.001
Plus HGS	0.743 (0.712–0.781)		

HGS, handgrip strength.

## 4 Discussion

By applying machine learning methods, the study found that HGS provided valuable information for mortality that is not captured by most traditional risk factors. and identified sex-specific thresholds that effectively identify individuals at high risk of mortality (<19 kg in women and < 32 kg in men). Moreover, the addition of HGS thresholds improved the prediction ability of an established office-based risk score, emphasizing its potential as a valid tool for risk discrimination in health screening setting.

Machine learning methods are able to handle complex and high-dimensional data, which allows for the extraction of significant features and the identification of novel relationships (12). In comparison to previous methods, such as logistic regression or traditional decision trees, random forest illustrated the prognostic value of HGS and its relative importance compared to other traditional risk factors. Furthermore, SHAP values were employed to uncover the “black box” of machine learning by quantifying the contribution of HGS to the model predictions. In the study, SHAP values plots intuitively displayed the nonlinear relationships and illustrated a clear threshold effect, which traditional methods like logistic regression might struggle to represent effectively.

A few studies indicated that HGS may provide similar and even stronger prognostic value than some traditional risk factors like BMI and blood pressure. In a study including 1,142,599 male adolescents followed up over 24 years, HGS was demonstrated to have similar predictive value for all-cause mortality to elevated BMI or blood pressure; the PURE study, including 139,691 adults followed up for 4 years, found that HGS was a stronger predictor of death than SBP (9, 18). The Feature importance plot of our random forest model confirmed that HGS as a significant predictor of mortality, providing more powerful prognostic information than most traditional risk factors. The explanation could be that HGS provides a more holistic representation of overall well-being and mortality risk. Firstly, HGS reflects multiple dimensions of physical health, including neuromuscular function and the level of frailty (19). Secondly, HGS is correlated with various age- and disease-related physiological processes, such as adiposity, insulin resistance, and elevated inflammation (20, 21). Furthermore, HGS can indirectly predict mortality risk by reflecting the presence and progression of chronic diseases (22).

For establishing thresholds for low HGS, previous studies have largely relied on data distribution. Some studies have defined thresholds at 2 or 2.5 standard deviations below the peak mean value across the life course; while others studies have established thresholds at the lower 20th or 25th percentile in the study population (5, 23–25). The utilization of these reference values has led to varying prevalence rates of low HGS, ranging from less than 10 to 25%, which could cause the underdiagnosis of high-risk individuals or over-recommendation of further assessments for patients. Therefore, HGS thresholds based on the specific levels of HGS values linked to health outcomes are needed to identify high-risk patients and avoid unnecessary waste of medical resources. In this study, we generated sex-specific thresholds within the random forest model, and used SHAP dependence plots to illustrate the nonlinear threshold effect. These HGS thresholds obtained from data-driven approaches identified 18.9% of the CHARLS population into high-risk group. The significant difference in baseline characteristics and mortality risk between groups further validated their ability to identify vulnerable people, and they may benefit from additional health assessments. Although previous study has shown that HGS enhances prediction of mortality based on age and sex, HGS thresholds were incorporated into a well-established office-based risk score to further validate their utility in screening setting (26). The results revealed an significant improvement in risk prediction (C index change 0.022), which surpasses the enhancements observed when adding HDL-C (C index change 0.007) and NT-proBNP (C index change 0.020) (27). This improvement in risk prediction suggests that adding HGS into screening would generate cost savings in healthcare system by reducing the number of patients who are introduced to further assessments.

The strength of this study is that it included a large and nationally representative sample size, thereby allowing for broad generalizability. Besides, this is the first study used machine learning methods to determine the predictive importance of HGS, establish thresholds for risk discrimination, and reveal the threshold effect. Significantly, the improvement of prediction when adding HGS thresholds into an established office-based risk score demonstrated the clinical utility of HGS in health screening settings. However, we also note several limitations. Firstly, the HGS data in our study were collected cross-sectionally, which hinders our ability to evaluate the impact of HGS changes on mortality over time. Secondly, the diagnosis of chronic diseases was self-reported physician-diagnosed, which may introduce some degree of bias. Thirdly, due to the unavailability of medical records and limited access to specific information on causes of death, our analysis of the association between HGS and cause-specific mortality remains limited in depth. However, these HGS thresholds still have clinical value in the large-scale health screening setting and may help save medical resources.

## 5 Conclusion

In conclusion, we successfully applied the machine learning method to illustrate the predictive importance of HGS for mortality and established reliable and practical sex-specific thresholds for identifying high-risk individuals. Our findings underscore the clinical utility of HGS in screening setting, particularly in resource-constrained regions like rural area and developing country.

## Data availability statement

The datasets presented in this study can be found in online repositories. The names of the repository/repositories and accession number(s) can be found in the article/Supplementary material.

## Ethics statement

The studies involving humans were approved by Ethical Review Committee at Peking University (No. IRB 00001052-11014). The studies were conducted in accordance with the local legislation and institutional requirements. The participants provided their written informed consent to participate in this study.

## Author contributions

HZ: Data curation, Validation, Writing – original draft. ZC: Formal analysis, Writing – review & editing. YuL: Data curation, Writing – original draft. YiL: Data curation, Writing – original draft. LG: Data curation, Writing – original draft. MX: Data curation, Formal analysis, Writing – original draft. BB: Formal analysis, Methodology, Writing – original draft. FL: Formal analysis, Writing – original draft. HM: Conceptualization, Funding acquisition, Supervision, Writing – review & editing. XY: Conceptualization, Supervision, Writing – review & editing. QG: Conceptualization, Supervision, Writing – review & editing.
