# Supplementary material for: Establishing thresholds of handgrip strength based on mortality using machine learning in a prospective cohort of Chinese population
Source: Front Med (Lausanne). 2023 Dec 1;10:1304181. doi: 10.3389/fmed.2023.1304181 (PMC10722261; doi:10.3389/fmed.2023.1304181)

**Supplementary TableS1. Baseline characteristics of participants stratified by sex‐specific thresholds of HGS**

| Characteristics | Training cohort (n=4733) | Validation cohort (n=2029) | P |
| --- | --- | --- | --- |
| Age, y | 58.8 (9.7) | 58.7 (9.6) | 0.524 |
| Female, n (%) | 2559 (54.1) | 1101 (54.3) | 0.903 |
| Married (vs others) | 3969 (83.9) | 1714 (84.5) | 0.549 |
| Rural (vs urban) | 1709 (36.1) | 773 (38.1) | 0.127 |
| Medical insurance, n (%) | 4435 (93.7) | 1892 (93.2) | 0.518 |
| Education |  |  | 0.226 |
| No formal education | 2134 (45.1) | 937 (46.2) |  |
| Primary school | 1086 (22.9) | 422 (20.8) |  |
| Middle or high school | 1451 (30.7) | 647 (31.9) |  |
| College or above | 62 (1.3) | 23 (1.1) |  |
| Smoking | 1873 (39.6) | 787 (38.8) | 0.563 |
| Drinking | 1267 (26.8) | 546 (26.9) | 0.419 |
| BMI | 23.9 (10.4) | 25.2 (54.9) | 0.286 |
| Blood pressure, mmHg |  |  |  |
| Systolic | 131.9 (30.4) | 132.7 (35.1) | 0.330 |
| Diastolic | 75.7 (11.9) | 76.0 (12.3) | 0.349 |
| Comorbidities, n (%) |  |  |  |
| Hypertension | 1234 (26.1) | 587 (28.9) | 0.016 |
| Diabetes | 318 (6.7) | 133 (6.6) | 0.846 |
| Cardiovascular disease | 603 (12.7) | 282 (13.9) | 0.210 |
| Stroke | 119 (2.5) | 54 (2.7) | 0.789 |
| Chronic lung disease | 453 (9.6) | 192 (9.5) | 0.925 |
| Chronic kidney disease | 260 (5.5) | 122 (6.0) | 0.429 |
| Liver disease | 165 (3.5) | 55 (2.7) | 0.116 |
| Cancers | 45 (1.0) | 13 (0.6) | 0.261 |
| Blood measurements |  |  |  |
| Hb, g/L | 14.5 (2.2) | 14.4 (2.0) | 0.329 |
| Hematocrit, % | 41.9 (6.1) | 41.6 (5.8) | 0.047 |
| MCV, fl | 90.9 (8.3) | 90.6 (8.3) | 0.094 |
| Total cholesterol, mg/dL | 193.5 (38.8) | 194.1 (38.7) | 0.571 |
| Triglycerides, mg/dL | 135.9 (107.5) | 133.2 (102.7) | 0.329 |
| HDL-C, mg/dL | 50.6 (15.1) | 51.1 (15.7) | 0.179 |
| LDL-C, mg/dL | 117.0 (35.4) | 117.8 (35.1) | 0.404 |
| eGFR, mL/min/1.73 m^2^ | 98.9 (25.5) | 97.8 (24.1) | 0.113 |
| HbA1c, mmol/mol | 5.3 (0.9) | 5.3 (0.8) | 0.983 |
| CRP, mg/L | 2.8 (7.5) | 2.7 (6.6) | 0.618 |
| Handgrip strength, kg | 31.9 (10.8) | 31.6 (10.9) | 0.311 |

HGS=handgrip strength; BMI=body mass index; Hb=hemoglobin; MCV=mean corpuscular volume; HDL=high density cholesterol; LDL=low density cholesterol; eGFR=estimated glomerular filtration rate; HbA1c= glycosylated hemoglobin; CRP=C-reactive protein.

**Supplementary Figure S1. Association between handgrip strength and age**


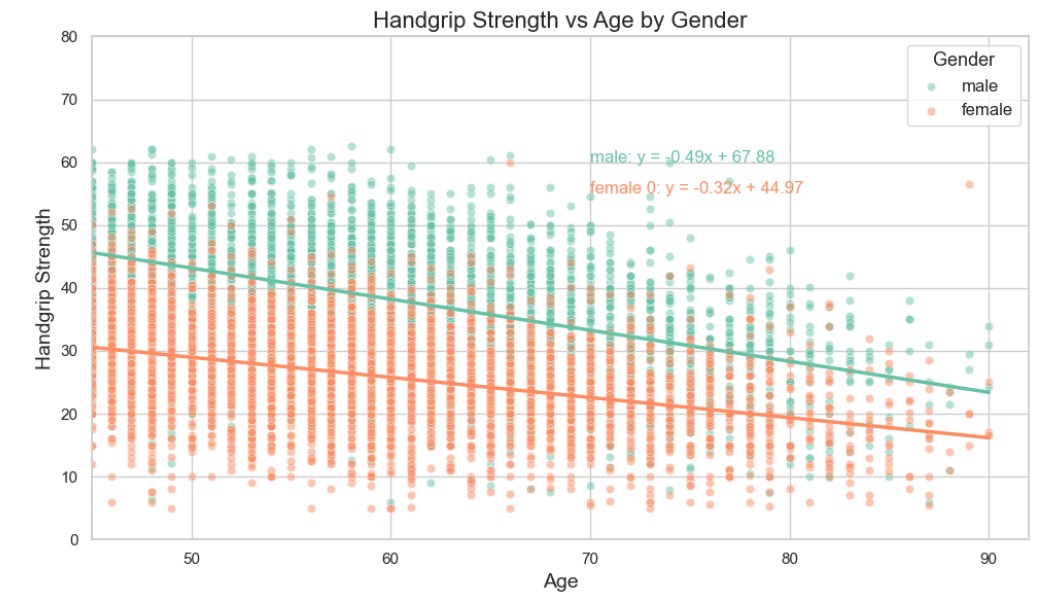


**Supplementary Figure S2. Thresholds of handgrip strength for different age range**


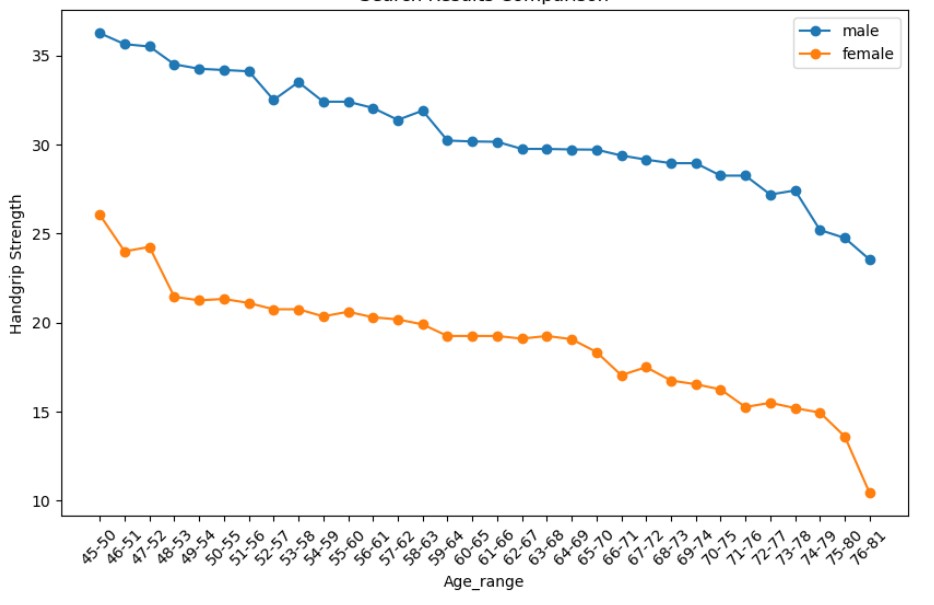


**Supplementary Figure S3. The Shapley Additive exPlanation summary plots of** **the random forest model.**


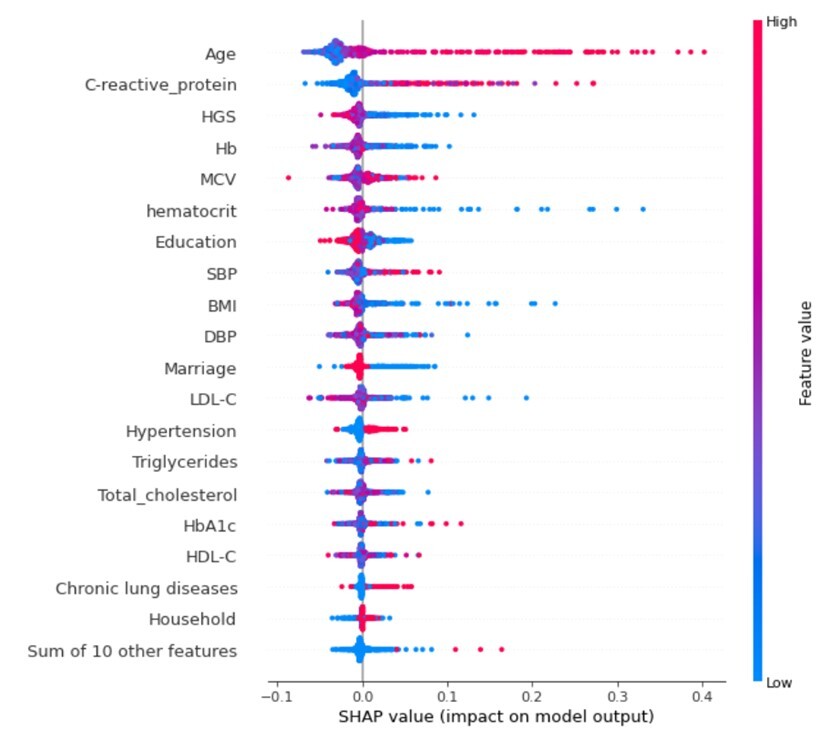

Supplement: Supplementary file 1 [file Data_Sheet_1.docx]
